# Cystatin SN promotes epithelial-mesenchymal transition and serves as a prognostic biomarker in lung adenocarcinoma

**DOI:** 10.1186/s12885-022-09685-z

**Published:** 2022-05-30

**Authors:** Jian Yang, Gaomeng Luo, Chang Li, Zhunlin Zhao, Sheng Ju, Qifan Li, Zhike Chen, Cheng Ding, Xin Tong, Jun Zhao

**Affiliations:** 1 Department of Thoracic Surgery, The First Affiliated Hospital of Soochow University, Suzhou, 215006 China; 2 Institute of Thoracic Surgery, The First Affiliated Hospital of Soochow University, Suzhou, China

**Keywords:** Lung adenocarcinoma, Epithelial-mesenchymal transition (EMT), Tumor immune microenvironment (TIME), Cystatin SN (CST1), Biomarker

## Abstract

**Background:**

Cystatins are a class of proteins that can inhibit cysteine protease and are widely distributed in human bodily fluids and secretions. Cystatin SN (CST1), a member of the CST superfamily, is abnormally expressed in a variety of tumors. However, its effect on the occurrence and development of lung adenocarcinoma (LUAD) remains unclear.

**Methods:**

We obtained transcriptome analysis data of CST1 from The Cancer Genome Atlas (TCGA) and GSE31210 databases. The association of CST1 expression with prognosis, gene mutations and tumor immune microenvironment was analyzed using public databases. Gene Ontology (GO), Kyoto Encyclopedia of Genes and Genomes (KEGG), and Gene Set Enrichment Analysis (GSEA) were performed to investigate the potential mechanisms of CST1.

**Results:**

In this study, we found that CST1 was highly expressed in lung adenocarcinoma and was associated with prognosis and tumor immune microenvironment. Genetic mutations of CST1 were shown to be related to disease-free survival (DFS) by using the c-BioPortal tool. Potential proteins binding to CST1 were identified by constructing a protein-protein interaction (PPI) network. Gene set enrichment analysis (GSEA) of CST1 revealed that CST1 was notably enriched in epithelial-mesenchymal transition (EMT). Cell experiments confirmed that overexpression of CST1 promoted lung adenocarcinoma cells migration and invasion, while knockdown of CST1 significantly inhibited lung adenocarcinoma cells migration and invasion.

**Conclusions:**

Our comprehensive bioinformatics analyses revealed that CST1 may be a novel prognostic biomarker in LUAD. Experiments confirmed that CST1 promotes epithelial-mesenchymal transition in LUAD cells. These findings will help to better understand the distinct role of CST1 in LUAD.

**Supplementary Information:**

The online version contains supplementary material available at 10.1186/s12885-022-09685-z.

## Background

Cysteine protease inhibitors were originally obtained from egg whites through affinity chromatography by Anastasi et al. [1]. Cystatins share sequence homology and a common tertiary structure of an alpha helix lying at the top of an anti-parallel beta strand [2]. Cystatins mainly suppress peptidase enzymes belonging to cysteine peptidase families C1 and C13 [3]. Interestingly, a growing number of experimental and clinical studies suggest that cystatin superfamily can affect all stages of cancer progression, including tumor growth, apoptosis, invasion, metastasis and angiogenesis [4]. There are four main inhibitory families in the cystatin superfamily, including the type I cystatins (Stefin family), type II cystatins (Cystatin family), type III cystatins (Kininogens family) and the type IV cystatins (Phytocystatin family) [5]. In these categories, Type II cystatins are the most investigated cystatins in cancer progression. Type II cystatins include a large number of homologous cysteine protease inhibitors which contain at least one cystatin domain [3]. Notably, S-type Cystatins (CST1, CST2, CST4), as members of Type II Cystatins, are proteins with unique roles, unlike other members. Existing studies suggest that they may play a role in promoting tumor progression [6, 7]. In particular, the role of CST1 in cancers is receiving increasing attention [8, 9].

CST1 mRNA contains three exons and two introns and it is translated into a protein containing 121 amino acid residues. Many studies have concluded that CST1 plays an important role in inflammation, tumorigenesis and tumor metastasis. For instance, in hepatocellular carcinoma, upregulation of cystatin SN promotes tumor progression and is predictive of a poor prognosis [10]. Moreover, Da-nian Dai et al. found that elevated CST1 expression promotes breast cancer progression [8]. In colorectal cancer, cystatin SN inhibits auranofin-induced cell death by autophagy and ROS regulation via its glutathione reductase activity [11]. Silencing cystatin SN abrogates cancer progression and stem cell properties in papillary thyroid carcinoma [12]. Furthermore, CST1 promotes gastric cancer migration and invasion by activating the Wnt pathway [13]. However, the roles and mechanisms of CST1 in LUAD have not been clarified. Non-small-cell lung cancer (NSCLC), the leading cause of human death worldwide, mainly includes adenocarcinoma, squamous cell carcinoma (LUSC) and large cell carcinoma [14]. Among them, LUAD is the most common clinical subtype of NSCLC, which is highly heterogeneous and aggressive [15]. At present, metastasis is still the leading cause of death in cancer patients, and approximately 90% of cancer deaths are caused by metastasis [16]. Epithelial-to-mesenchymal transition is a key event in the transformation of early tumors into advanced malignant tumors and is closely related to the invasion and metastasis of tumor cells [17]. In this research, we comprehensively explored the expression, prognostic value and mutation characteristics of CST1 in LUAD. By constructing PPI networks of CST1 and its co-expressed genes, the function of CST1 was probed. In addition, we evaluated the potential association of CST1 with EMT in LUAD through functional analysis and experimental verification.

## Methods

### Data collection

The RNA expression and clinicopathological data of TCGA and GTEx were downloaded from the UCSC Xena database (https://xenabrowser.net/datapages/) [18]. GSE31210 was downloaded from the GEO database. Protein interaction networks were constructed by the GeneMANIA online tool (https://genemania.org/) [19] and STRING database (https://cn.string-db.org/) [20].

### GEPIA

The GEPIA database is a newly developed web-based online tool [21]. (http://gepia.cancer-pku.cn/) GEPIA can provide critical interaction and customization functions, such as differential expression analysis of tumor and normal RNAs, mapping based on cancer types or different pathological stages, patient survival analysis, correlation analysis, dimensional reduction analysis, and similar genetic testing based on data from the GTEx project and TCGA.

### The cBioPortal

The cBioPortal for cancer genomics is an open online resource for the interactive exploration of a wide range of cancer genomics databases. The cBioPortal significantly reduces access barriers between complex genomic data and cancer researchers, facilitating rapid, intuitive, and high-quality access to molecular profiles and the clinical prognostic relevance of large-scale cancer genomics projects [22]. We selected 12 LUAD datasets for further analysis. (https://www.cbioportal.org/).

#### Kaplan–Meier Plotter database

We analyzed the prognostic value of CST1 in lung cancer using Kaplan–Meier Plotter (https://kmplot.com/analysis/), an online database of gene expression and survival data from 1925 clinical lung cancer patients (719 LUAD patients) [23]. Patient samples were divided into two groups (high and low expression) based on median expression to analyze the overall survival (OS) and first progression survival (FP) with HRs with 95% CIs and Log-rank *p* values.

#### PrognoScan database

The correlation between CST1 expression and prognostic parameters, such as OS and relapse-free survival (RFS) rates, can be investigated using PrognoScan in a large open cancer microarray dataset [24]. Hazard ratios with 95% confidence intervals were calculated. The database threshold was a Cox *P* value< 0.05. (http://dna00.bio.kyutech.ac.jp/PrognoScan/index.html).

### GO and KEGG pathway enrichment analyses and GSEA

The biological function of CST1 in LUAD was assessed by GO and KEGG enrichment analyses [25]. Biological processes, cellular components and molecular functions relevant to CST1 were identified by GO, a mighty bioinformatics tool [26]. Potential mechanisms of CST1 were investigated using GSEA. GSEA can be used to determine whether a set of a priori defined genes differs significantly between two biological states. GO and KEGG analyses were performed using the R package ClusterProfiler [27]. GSEA was performed using GSEA software 4.1.0.

### Cell culture and specimens

BEAS2B, HBE and human LUAD cell lines (A549, H1299 and H1650) from the Cell Bank of the Chinese Academy of Sciences were cultured in RPMI 1640 medium (Shanghai Yuanpei Company) supplemented with 10% fetal bovine serum in a 5% CO2 humidified incubator at 37 °C [28]. A total of 31 LUAD tissues and corresponding paraneoplastic tissues were obtained from patients that provided informed consent at the First Affiliated Hospital of Soochow University (Suzhou, China). These specimens were immediately stored at − 80 °C until further processed. This work was authorized by the Ethics Committee of Soochow University.

### Western blot assessment

Cells were hydrolyzed in RIPA buffer with a mixture of protease inhibitors and phosphatase inhibitors. Protein products were isolated by SDS–PAGE and transferred onto nitrocellulose membranes. We cut the membranes according to the molecular weight of the target proteins. The membranes were then blocked in TBST buffer with 3.0% BSA for 1 hour and incubated at 4 °C with primary antibodies overnight. The membranes were rinsed three times with TBST buffer and incubated at room temperature for 2 hours with the corresponding secondary antibody. Protein detection was performed using an enhanced chemiluminescence system. The expression of proteins of interest was normalized to that of β-actin. The following primary antibodies used in WB analysis: anti-mouse snail, anti-mouse Vimentin, anti-mouse E-cadherin, and anti-mouse N-cadherin (Cell Signaling Technology, Danvers, MA, USA); anti-rabbit CST1, anti-mouse GAPDH, and anti-mouse β-actin (ABclonal, Biotechnology Co. Ltd. Wuhan, China); and anti-rabbit or anti-mouse secondary antibodies (Beyotime Biotechnology Co. Ltd. Shanghai, China). Relative protein expression was normalized to that of β-actin. The same set of lysate samples were run in parallel (sister) gels to test different proteins (same for all figures).

### RNA extraction and qRT–PCR assays

Total RNA was extracted using a standard TRIzol RNA extraction protocol [29]. Synthesis of cDNA with reverse transcriptase was conducted using the BeyoRT™ II First Strand cDNA Synthesis Kit. The expression of CST1 mRNA was detected using qRT–PCR. The expression of CST1 was normalized to that of GAPDH. Relative mRNA levels were calculated using the ΔΔCt method [30]. Gene expression levels were calculated relative to that of GAPDH. The following primers were used: CST1 (Forward Primer: GGGTGGCATCTATAACGCAG; Reverse Primer: CTGTTGCCTGGCTCTTAGT); GAPDH (Forward Primer: TGACTTCAACAGCGACACCA; Reverse Primer: CACCCTGTTGCTGTAGCCAAA).

### Overexpression plasmid and RNA interference

The coding sequence of CST1 (NM_001898.3) was amplified with the appropriate primers (forward primer: ATAGAAGATTCTAGAATGGCCCAGTATCTG; reverse primer: GTATGGGTAGGATCCGGATTCTTGACACCT), and the pcDH-CST1-HA plasmid was generated by using the endonucleases XbaI/BamhI. siRNAs for CST1 were synthesized by GenePharma (Shanghai, China). The sequences of the siRNAs were as follows: si-CST1–1 (GUGGCAUCUAUAACGCAGATTUCUGCGUUAUAGAUGCCACTT) and si-CST1–2 (GGUACUAAGAGCCAGGCAATTUUGCCUGGCUCUUAGUACCTT). The A549 and H1299 cell lines were transfected with the aforementioned plasmid or siRNAs (100 pmol) using with Lipofectamine 2000 or 3000 (Invitrogen) [31].

### Wound-healing migration assay

A549 cells and H1299 cells were inoculated and cultured in 6-well plates until they reached 95% confluence. The cells were then scraped with the tip of a sterilized straw and cultured in serum-free media for 24 hours [32]. The cells were photographed under a microscope in five random areas. ImageJ Launcher software was used to analyze the distances of cell migration to scratched areas. Each wound healing experiment was repeated in triplicate.

### Transwell assays

Transwell panels were used for the Transwell test (BD Biosciences). Briefly, A549 cells or H1299 cells with 1% FBS medium were added in each upper chamber and 20% FBS medium was placed as chemoattractant in each lower chamber. The chamber was removed after 24 hours of incubation in a 37 °C incubator, and the upper surface was cleaned with swabs. The cells invading the lower layer were stained with 1% crystalline violet. Cells from three microscope images were photographed and counted [33]. The results were affirmed through three repeat trials.

### Immunofluorescence

For immunofluorescence assay, cells seeded on coverslips which were washed with 1 × PBS and fixed with 4% PFA for 20 minutes. Then cells were permeabilized with 0.5%Triton X-100 and blocked with 5% BSA for 30 minutes. After three washes with 1 × PBS, the cells were incubated with the first antibodies at 4 °C overnight and then followed by one-hour staining with fluorescent secondary antibodies (Abcam, USA) and counterstained with DAPI [34].

## Results

### CST1 expression is increased in LUAD patients and associated with clinical parameters

**Fig. 1:**
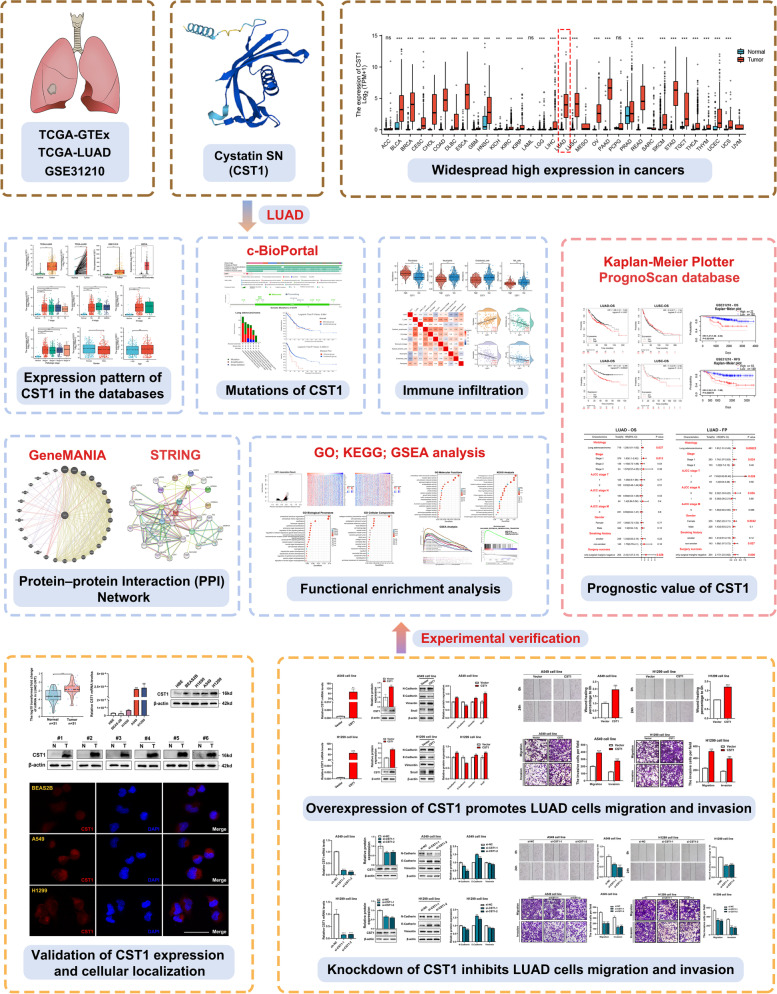
Flowchart of construction and analysis of CST1

**Fig. 2:**
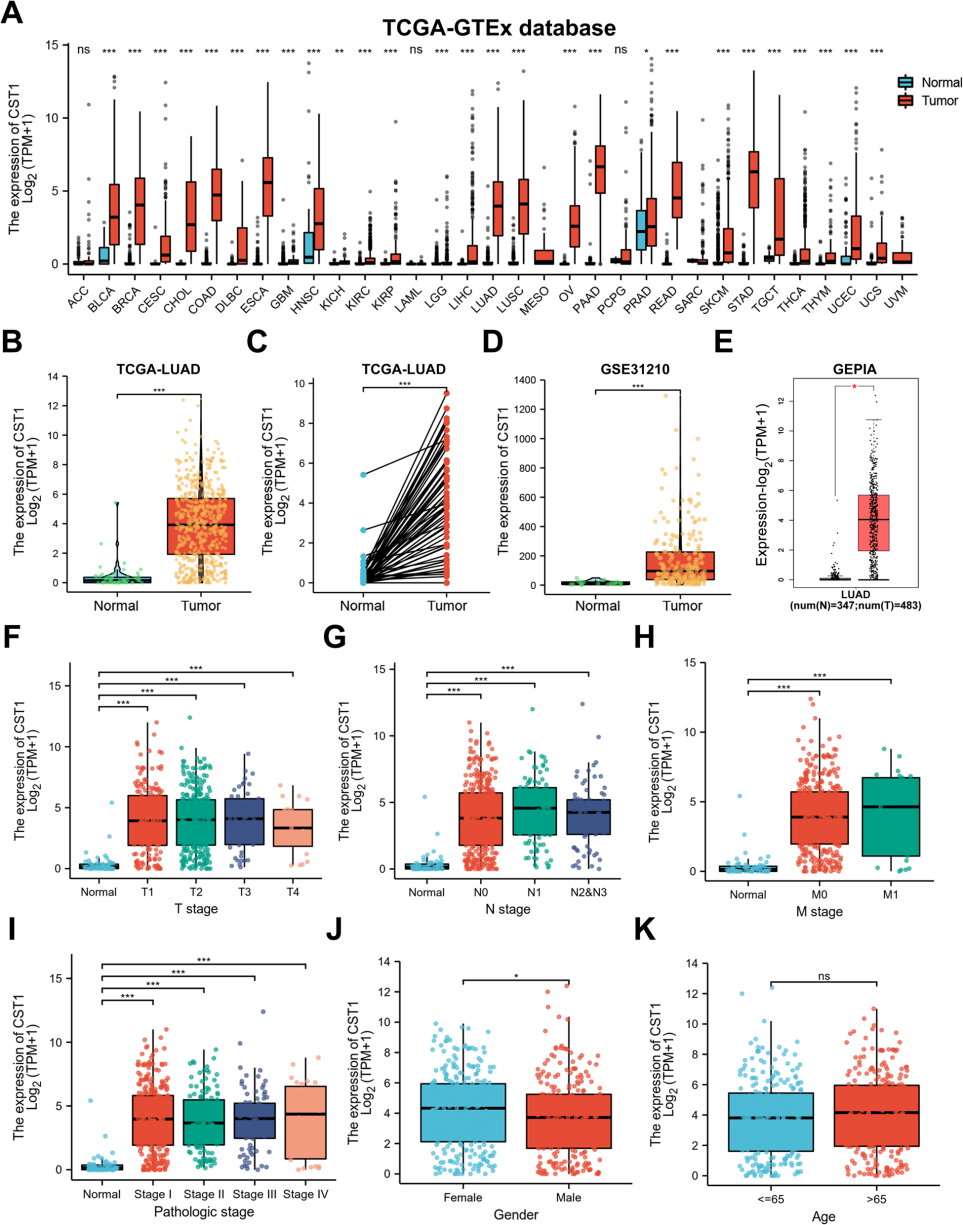
Expression of CST1 in lung adenocarcinoma. **a** CST1 expression in different types of cancers was investigated in the TCGA-GTEx database. **b** Increased expression of CST1 in LUAD compared to normal tissues in the TCGA database. **c** Statistical analyses of CST1 expression in 58 pairs of LUAD tissues and adjacent normal tissues. **d** CST1 expression in LUAD was examined by using the GSE31210 database. **e** Analysis of CST1 expression in LUAD and adjacent normal tissues in the GEPIA database. **f**-**i** CST1 expression in different pathological stages and TNM stages in the TCGA database. **j**-**k** CST1 expression in different gender and age in the TCGA database. *p < 0.05, **p < 0.01 ***p < 0.001

**Fig. 3:**
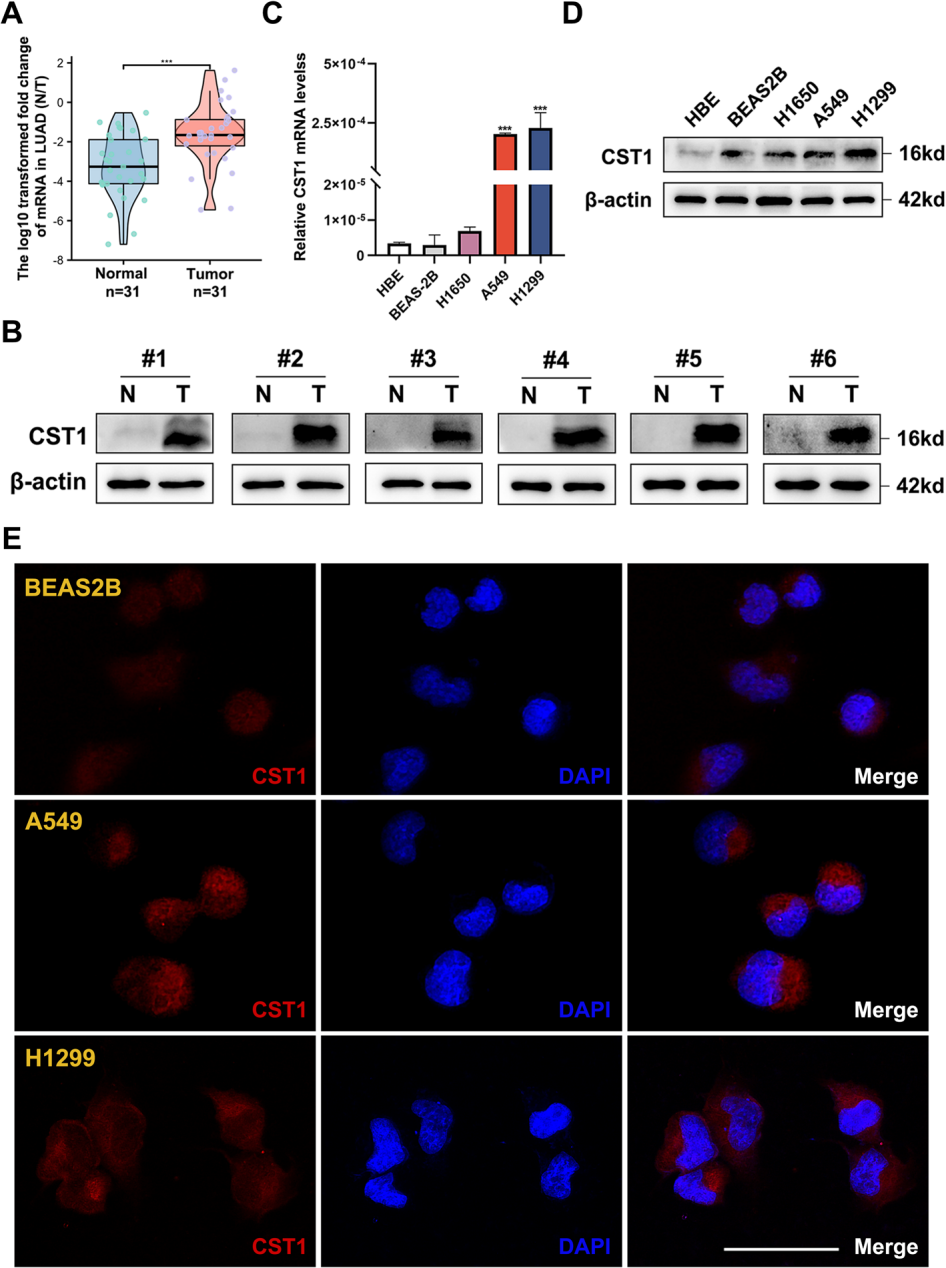
Validation of CST1 expression and cellular localization. **a** Relative expression of CST1 in 31 pairs of LUAD tissues and adjacent normal tissues. **b**-**c** Relative CST1 mRNA and protein levels in HBE, BEAS2B, H1650, A549, H1299 cell lines. **d** The WB results of CST1 in representative 6 pairs of LUAD tissue and adjacent normal tissues. **e** Localization of CST1 in BEAS2B, A549 and H1299 cells. *p < 0.05, **p < 0.01 ***p < 0.001

The flow diagram of our present study is illustrated in Fig. 1. CST1 is generally highly expressed in the TCGA-GTEx pan-cancer database (Fig. 2a), indicating that CST1 may function as an oncogene in cancers. We used the TCGA database (Fig. 2b, c), GSE31210 (Fig. 2d) and GEPIA database (Fig. 2e) to analyze the expression level of CST1 in LUAD and found that CST1 was significantly over-expressed in tumors. Then, we used the TCGA database to analyze the expression of CST1 at different pathological and TNM stages, suggesting that CST1 is associated with clinical parameters (Fig. 2f-i). In addition, the expression differences of CST1 in different gender and age were compared. We found that the expression levels of CST1 in female patients were slightly higher than that in male patients, but there was no statistical difference in age (Fig. 2j-k). To verify the database results, 31 pairs of paracancerous and cancerous tissues were subjected to qRT–PCR analysis, and CST1 expressions were found to be higher in cancer tissues (Fig. 3a). The representative WB results of 6 pairs of tissue specimens were shown in Fig. 3b. In addition, we verified this finding in the HBE, BEAS2B, H1650, A549, and H1299 cell lines (Fig. 3c, d). Among them, HBE and BEAS2B cells were primary control cells while H1650, A549 and H1299 cells were lung adenocarcinoma cells. The results showed CST1 was dramatically overexpressed in LUAD cells, especially A549 and H1299 cell lines, paving the way for their selection for further experimental verification. Furthermore, we investigated the cellular localization of CST1 in BEAS2B, A549 and H1299 cells. Immunofluorescence results revealed that CST1 was primarily located in the cytoplasm of normal lung epithelial cells and lung adenocarcinoma cells, and the staining pattern was vesicular. In addition, CST1 was strongly expressed in A549 and H1299 cells, but less in BEAS2B cells (Fig. 3e).

### Increased CST1 expression correlates with poor prognosis in LUAD patients

**Fig. 4:**
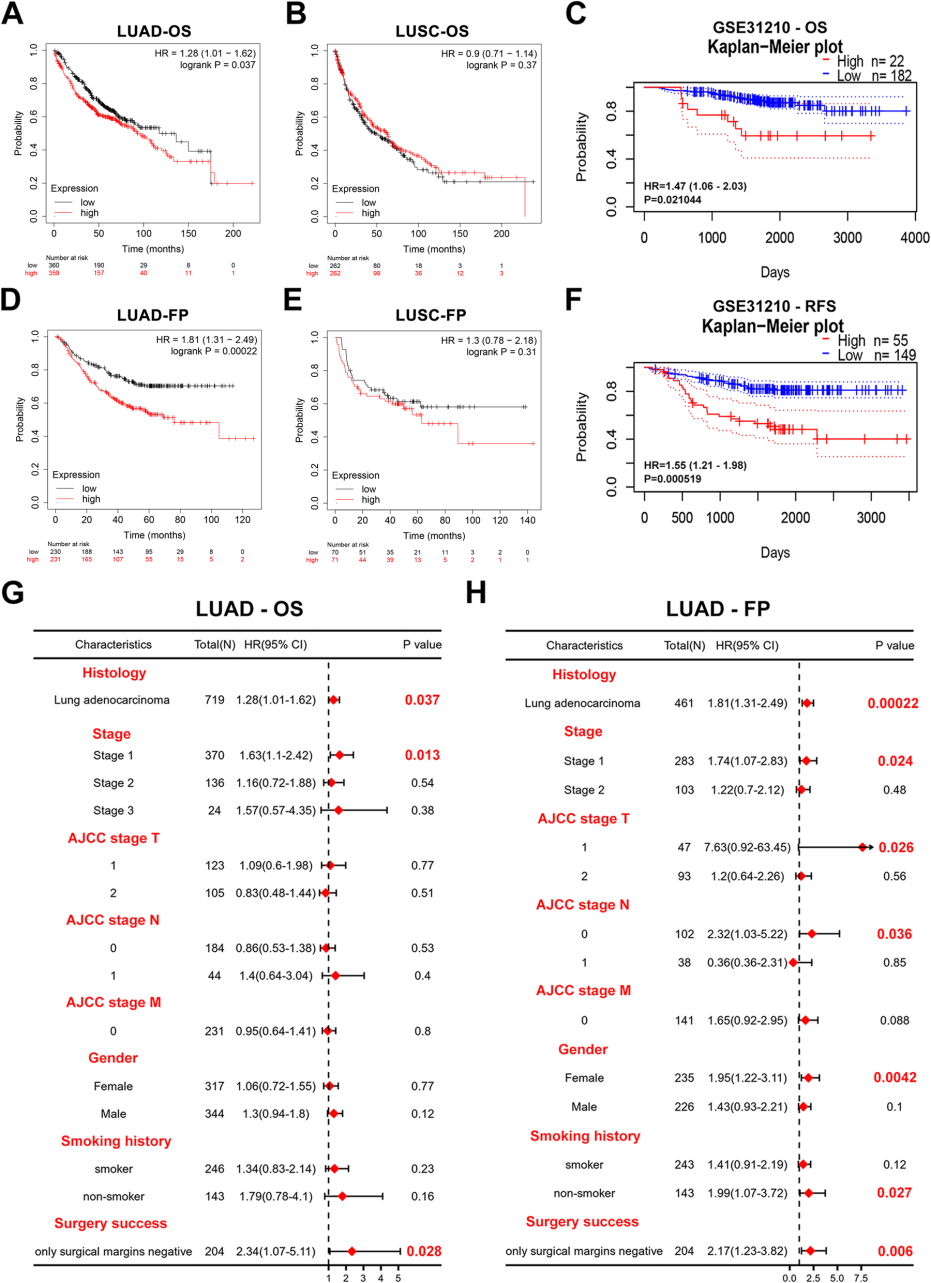
Prognostic value of CST1. **a**, **b**, **d**, **e** Kaplan–Meier survival curves showed the impact of CST1 on overall survival (OS) and first progression (FP) survival in lung adenocarcinoma and lung squamous cell carcinoma patients. **c**, **f** Overall survival and relapse-free survival curves of CST1 were shown using the PrognoScan database. **g**-**h** Forest plots showed the prognostic value (OS, FP) of CST1 based on various clinicopathological features in LUAD patients

We used the Kaplan–Meier Plotter database to carry out survival analysis based on the overall survival (OS) (Fig. 4a, b) and first progression survival (FP) (Fig. 4d, e) of LUAD and LUSC patients, respectively. We found that for the OS and FP of LUAD patients, high expression of CST1 was correlated with poor prognosis (*P* < 0.05). However, the results were not statistically significant in LUSC. To confirm this result, survival analysis was performed with the GSE31210 (LUAD) database (Fig. 4c, f), and high expression of CST1 was correlated with poor prognosis in terms of OS and relapse-free survival (RFS) (*P* < 0.05).

### Prognostic value of CST1 based on various clinicopathologic features

To better comprehend the prognostic value and possible mechanism of CST1 in LUAD, Kaplan-Meier Plotter was used to analyze the expression levels of CST1 and impacts of each factor on prognosis of OS and FP in LUAD. The stage 1 and only surgical margin negativity parameters were correlated with poor OS in LUAD (Fig. 4g). Regarding the FP of LUAD patients, the parameters stage 1, AJCC stage T1, AJCC stage N1, female sex, nonsmoker, and only negative surgical margins were associated with poor prognosis (Fig. 4h). These results suggested that mRNA expression of CST1 had prognostic value for LUAD.

#### Genetic alteration and PPI network of CST1 in LUAD

**Fig. 5:**
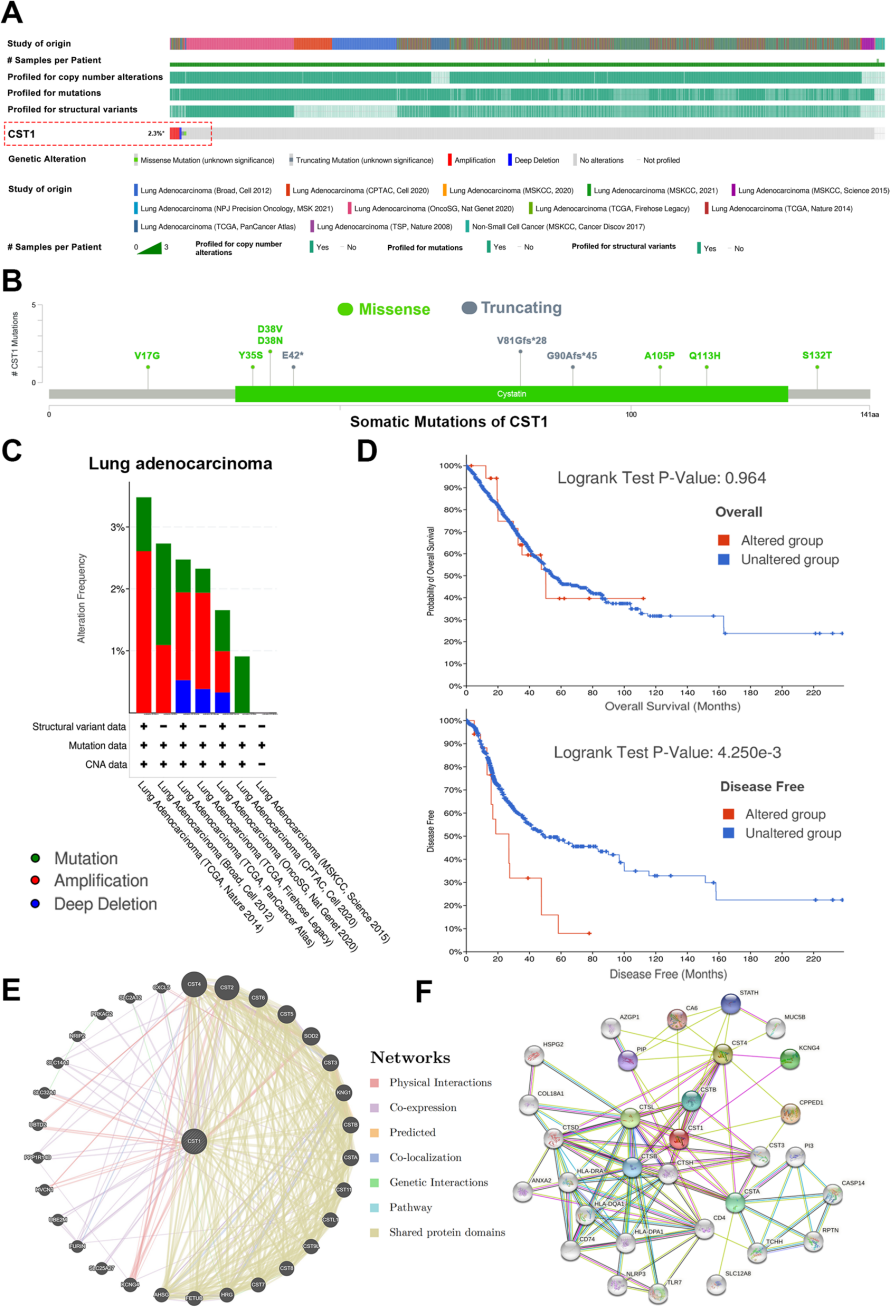
Genetic mutations and PPI networks of CST1 in LUAD. **a** OncoPrint of c-BioPortal showed the mutation proportions of CST1 in 4309 LUAD samples. **b** Somatic mutations of CST1. **c** Mutation types of CST1 in 7 databases. **d** Prognostic correlations between CST1 and mutations. **e** PPI network of their interacting genes visualized by GeneMANIA. **f** Top 30 PPI network made by STING database

The genetic mutation of CST1 in LUAD was analyzed by using the c-BioPortal online tool. We selected 4309 samples (lung adenocarcinoma) from 12 databases in the c-BioPortal (Fig. 5a**)**. By analyzing these samples, we found an overall mutation rate of 2.3% for CST1, with DNA copy number amplification being the most common mutation pattern (Fig. 5c**)**. In addition, we identified a total of 10 somatic mutation sites in CST1. Seven of the sites were mutated in a missense way, and three were mutated in a truncating way (Fig. 5b**)**. Furthermore, we investigated the prognostic differences between patients in the mutant and non-mutant groups. Results showed no significant difference in overall survival, but in disease-free survival, patients with mutations had significantly worse survival than those without mutations (Fig. 5d**)**. These suggested that mutations in CST1 may influence tumor progression and, consequently, patients’ outcome. Remarkably, we also used GeneMANIA and STRING databases to explore genes that potentially interact with CST1, and construct protein-protein interaction networks. GeneMANIA and STRING datasets displayed the top 30 interacting genes of CST1, respectively (Fig. 5e, f**)**. The protein networks include seven types of connections: Physical Interactions, Co-expression, Predicted, Co-localization, Genetic Interactions, Pathway, Shared protein domains. Interestingly, we found a large number of CST superfamily members in the protein network, which suggested that CST1 may play a role in promoting tumor progression in combination with other members of the CST superfamily.

#### Tumor immune microenvironment of CST1 in LUAD

**Fig. 6:**
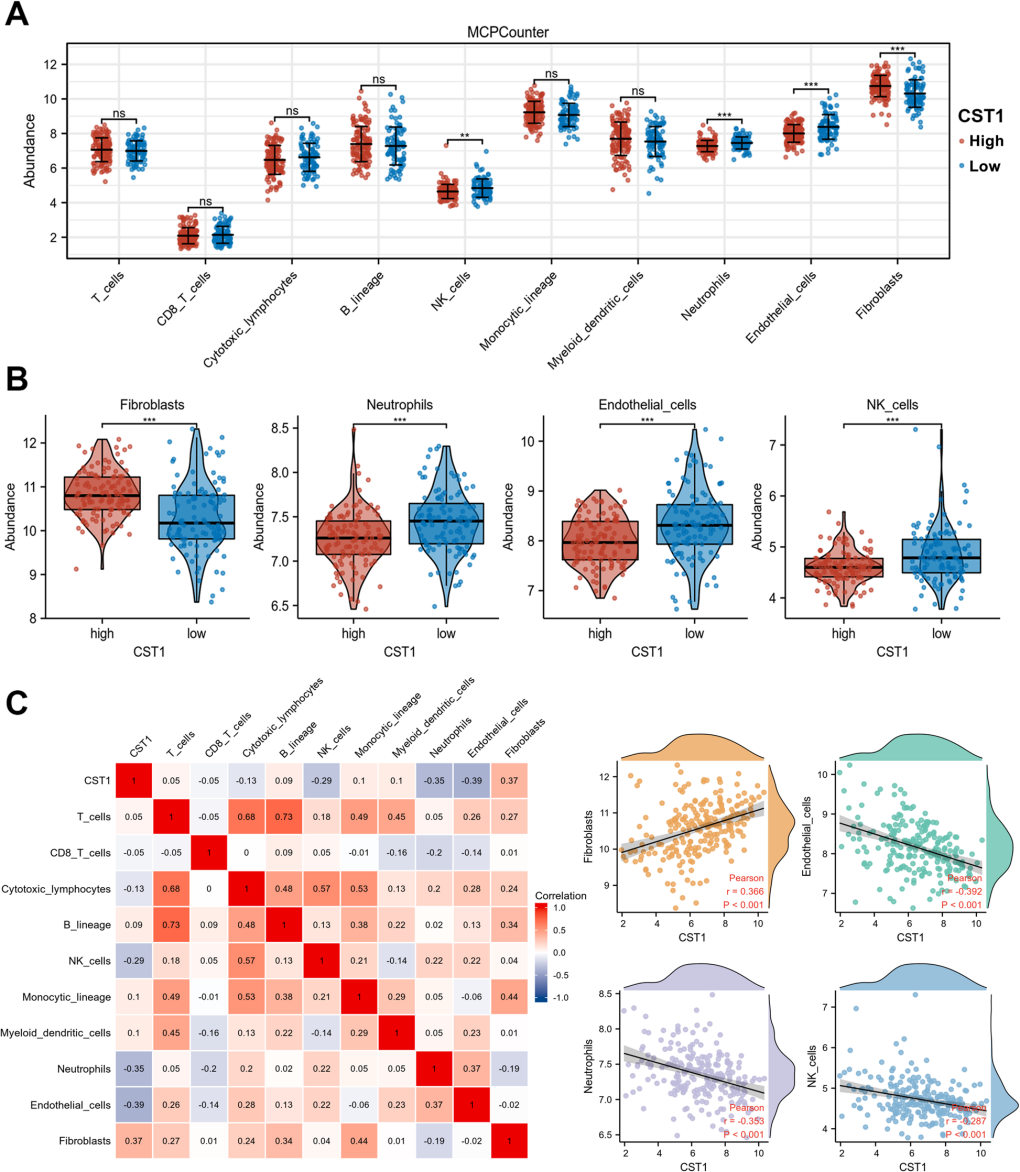
Correlation of tumor microenvironment and CST1 expression in LUAD patients. **a**, **b** Differences in infiltration abundance of 10 immune cell types with high and low expression groups of CST1. **c** Relationships among infiltration levels of 10 immune cell types and CST1 expression profiles by Pearson’s analysis

Lung adenocarcinoma has an extremely complex tumor immune microenvironment, which is closely associated with tumor progression and metastasis. Therefore, it is relevant to explore whether CST1 can contribute to tumor progression by affecting the tumor microenvironment. We analyzed the tumor immune microenvironment of CST1 by using the GSE31210 database. Based on the gene expression profile, immune infiltrating cells scores were calculated for each sample using the MCPCounter algorithm via the R package IOBR. As shown in Fig. 6a, b, the infiltration levels of Fibroblasts, NK cells, Neutrophils, and Endothelial cells were significantly different between the high and low CST1 expression groups. Tumor-associated fibroblasts (CAFs) were more abundant in the CST1 high expressed group, whereas endothelial cells, neutrophils, and NK cells were less abundant in the CST1 high expressed group. In addition, we used the Pearson analysis to investigate the correlation between CST1 and infiltration abundance of these 10 cell types. Notably, we found that CST1 expression levels were positively correlated with Fibroblasts, while negatively correlated with NK cells, Neutrophils, and Endothelial cells (Fig. 6c). These results suggested that CST1 may play a pro-tumor role by affecting the immune and stromal microenvironments.

#### GO and KEGG analyses of genes co-expressed with CST1 in LUAD

**Fig. 7:**
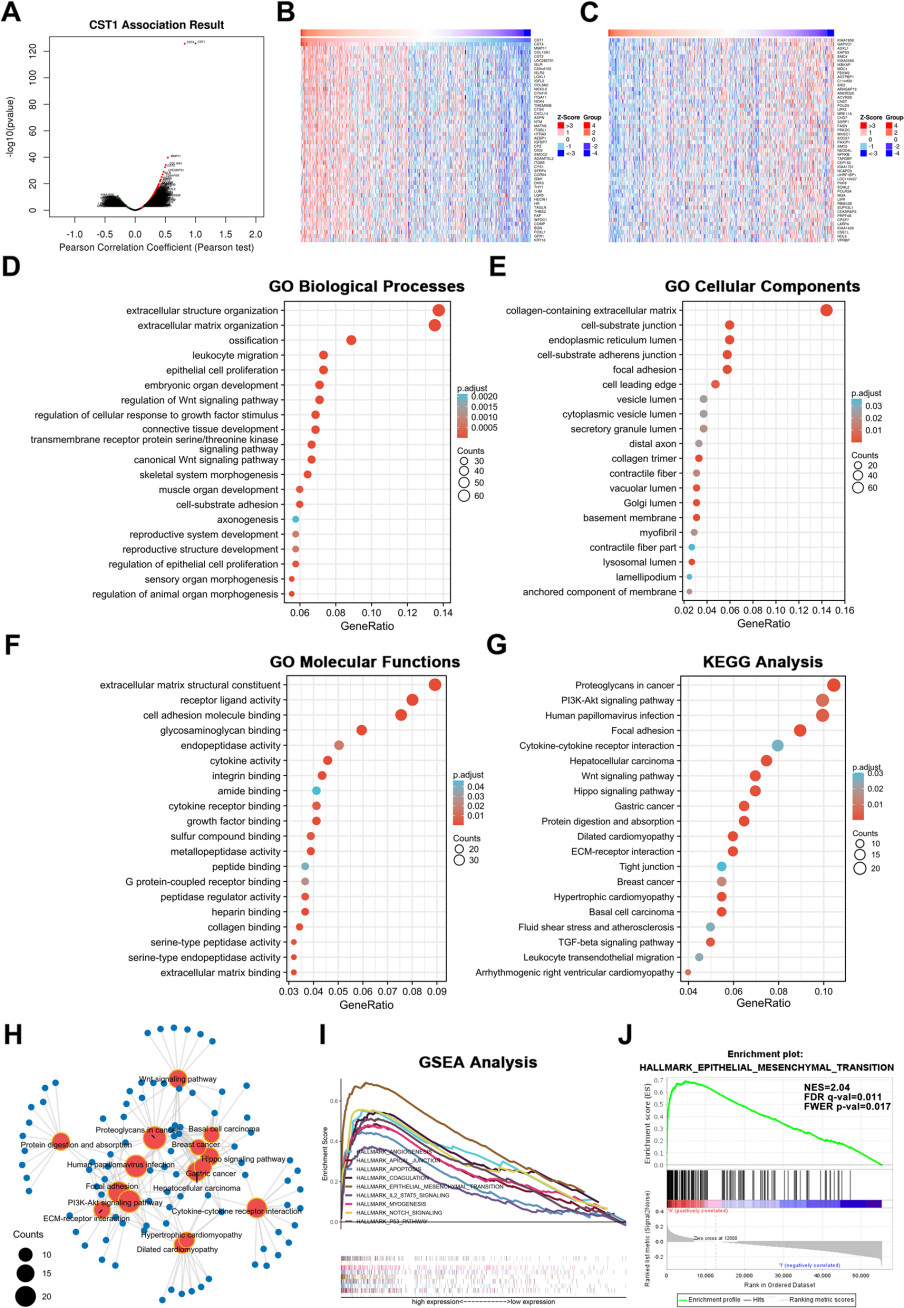
GO, KEGG and GSEA enrichment analysis for CST1. **a** Volcano plot of CST1 co-expressed genes was made by Linkedomics. **b**-**c** Top 50 positively and top 50 negatively correlated genes of CST1. **d** Top 20 enrichment terms in BP categories in LUAD. **e** Top 20 enrichment terms in CC categories in LUAD. **f** Top 20 enrichment terms in MF categories in LUAD. **g** Top 20 KEGG enrichment pathways in LUAD. **h** Visual network of Top 15 KEGG enrichment pathways in LUAD. **i**-**j** GSEA enrichment analyses of differential expressed genes indicating an association of CST1 with EMT

We used the Linkdomics database to identify genes co-expressed with CST1 in LUAD. The gene denoted by the red dot was positively associated with CST1, while that denoted by the green dot was negatively associated with CST1 (*P* < 0.01) (Fig. 7a). The top 50 genes positively and negatively correlated with CST1 are listed in Fig. 7b and c, respectively. Subsequently, GO (Additional file 1: Table S1) and KEGG enrichment analyses of the top 500 genes that were positively correlated with CST1 expression in the TCGA database were performed (Additional file 2: Table S2). Figure 7d showed the enrichment analysis of the top 20 biological processes, and the results revealed that CST1 is mainly related to extracellular structure organization, extracellular matrix organization, ossification, epithelial cell proliferation, leukocyte migration, and regulation of the Wnt signaling pathway. Figure 7e showed the accumulation analysis of the top 20 cellular components, and the results revealed that CST1 is mainly associated with collagen-containing extracellular matrix, the endoplasmic reticulum lumen, cell-substrate junctions, focal adhesion and adherens junctions. Figure 7f showed the enrichment analysis of the top 20 molecular functions, and the results revealed that CST1 is mainly related to extracellular matrix structural constituents, receptor ligand activity, cell adhesion molecule binding, glycosaminoglycan binding, and endopeptidase activity. Then, we performed KEGG enrichment analysis of CST1, and the results revealed that CST1 is mainly related to EMT (Fig. 7g). For example, the PI3K-Akt signaling, focal adhesion, Wnt signaling, ECM-receptor interaction, tight junction, and TGF-beta signaling pathways were identified. Furthermore, the KEGG visual network of CST1 was constructed and shown in Fig. 7h.

### CST1 was enriched for EMT in LUAD as determined by GSEA

The molecular mechanisms of CST1 in LUAD patients were further investigated by GSEA. According to the CST1 median expression level, the gene expression data were divided into high and low expression groups, and the gene sets were arranged 1000 times for each analysis. Throughout this process, we considered the expression level of CST1 to be the phenotype. Phenotypic enrichment pathways were classified according to the p.adjust values and normalized enrichment scores (NESs). We chose the hallmark pathway, and the GSEA results showed that multiple gene sets were enriched in LUAD, including those related to epithelial-mesenchymal transition, the P53 pathway, coagulation, NOTCH signaling, apical junctions, myogenesis, apoptosis, angiogenesis, and IL2_STAT5 signaling (Additional file 3: Table S3). These results strongly indicated the involvement of CST1 in the regulation of EMT in LUAD (Fig. 7i, j).

### Overexpression of CST1 promotes LUAD cell migration and invasion

**Fig. 8:**
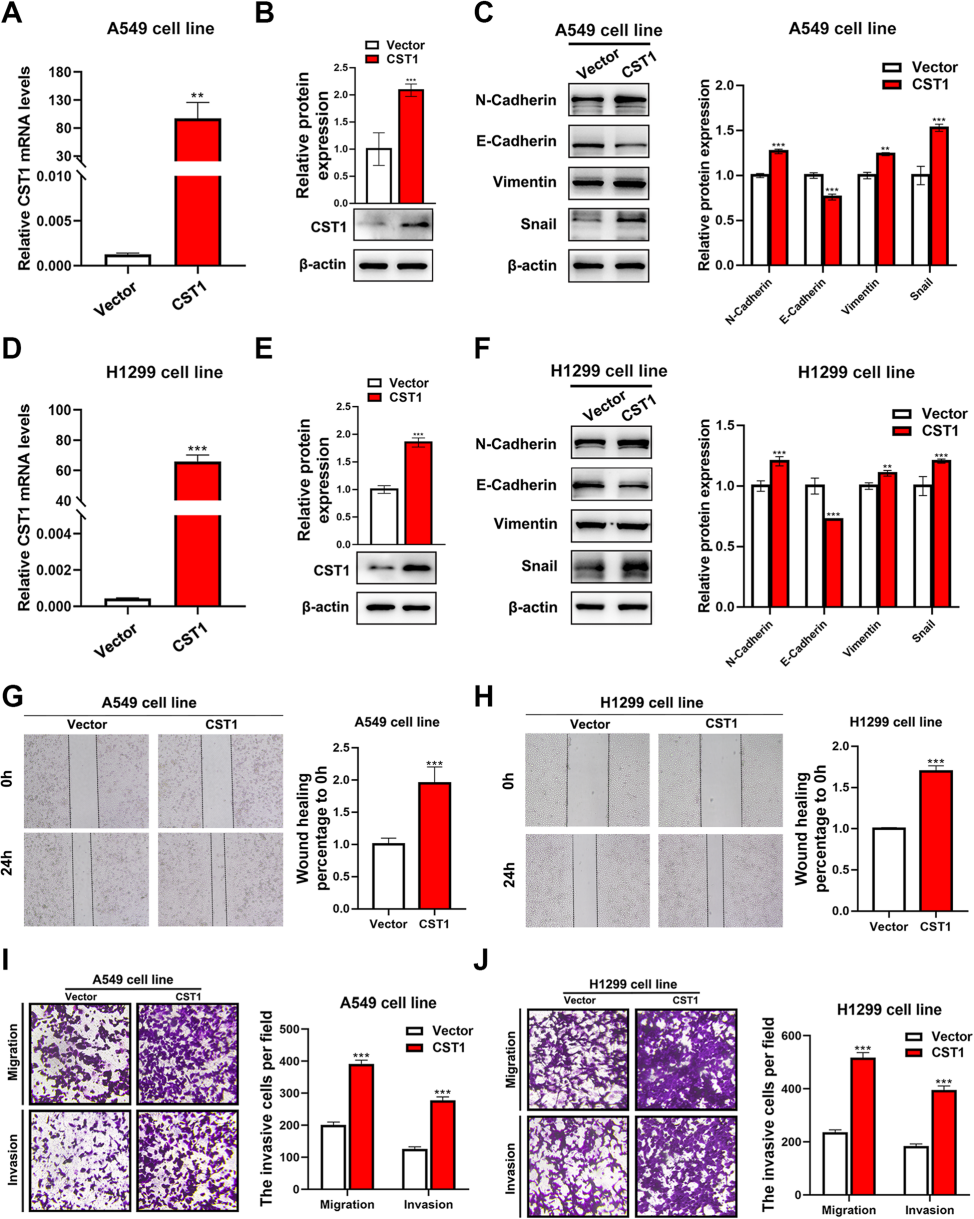
Overexpression of CST1 promotes EMT in A549 and H1299 cells. **a**, **d** The mRNA levels of CST1 in A549 and H1299 cells overexpressing CST1 were detected by q-PCR. **b**, **e** The protein levels of CST1 in A549 and H1299 cells overexpressing CST1 were detected by western blotting. **c**, **f** The expression of EMT markers was detected by western blotting. **g**, **h** Wound-healing migration assays of A549 and H1299 cells overexpressing CST1 as well as control cells, which were treated as described above. **i**-**j** A549 and H1299 cells overexpressing CST1 were allowed to migrate through an 8-μM-pore size transwell insert. Overexpression of CST1 inhibits the migration and invasion abilities of A549 and H1299 cells. Migrated and invaded cells were stained and counted in at least three microscopic fields. *p < 0.05, **p < 0.01 ***p < 0.001. (The blots were cutted prior to hybridisation with antibodies)

We overexpressed CST1 in A549 and H1299 cells and then detected the mRNA levels of CST1 using qRT–PCR (Fig. 8a, d). The protein level of CST1 was detected by western blot (Fig. 8b, e). EMT-associated markers in cell lines overexpressing CST1 were measured, revealing increased levels of N-cadherin, Vimentin and Snail and a decreased level of E-cadherin (Fig. 8c, f). The migration capacity of CST1-overexpressing cell lines was determined by the scratch method, revealing the enhanced migration of A549 and H1299 cells overexpressing CST1 compared to the vector control cell lines (Fig. 8g, h). The Transwell results revealed that the migration and invasion abilities of the above-mentioned cell lines were also enhanced (Fig. 8i, j).

### Knockdown of CST1 inhibits LUAD cell migration and invasion

**Fig. 9:**
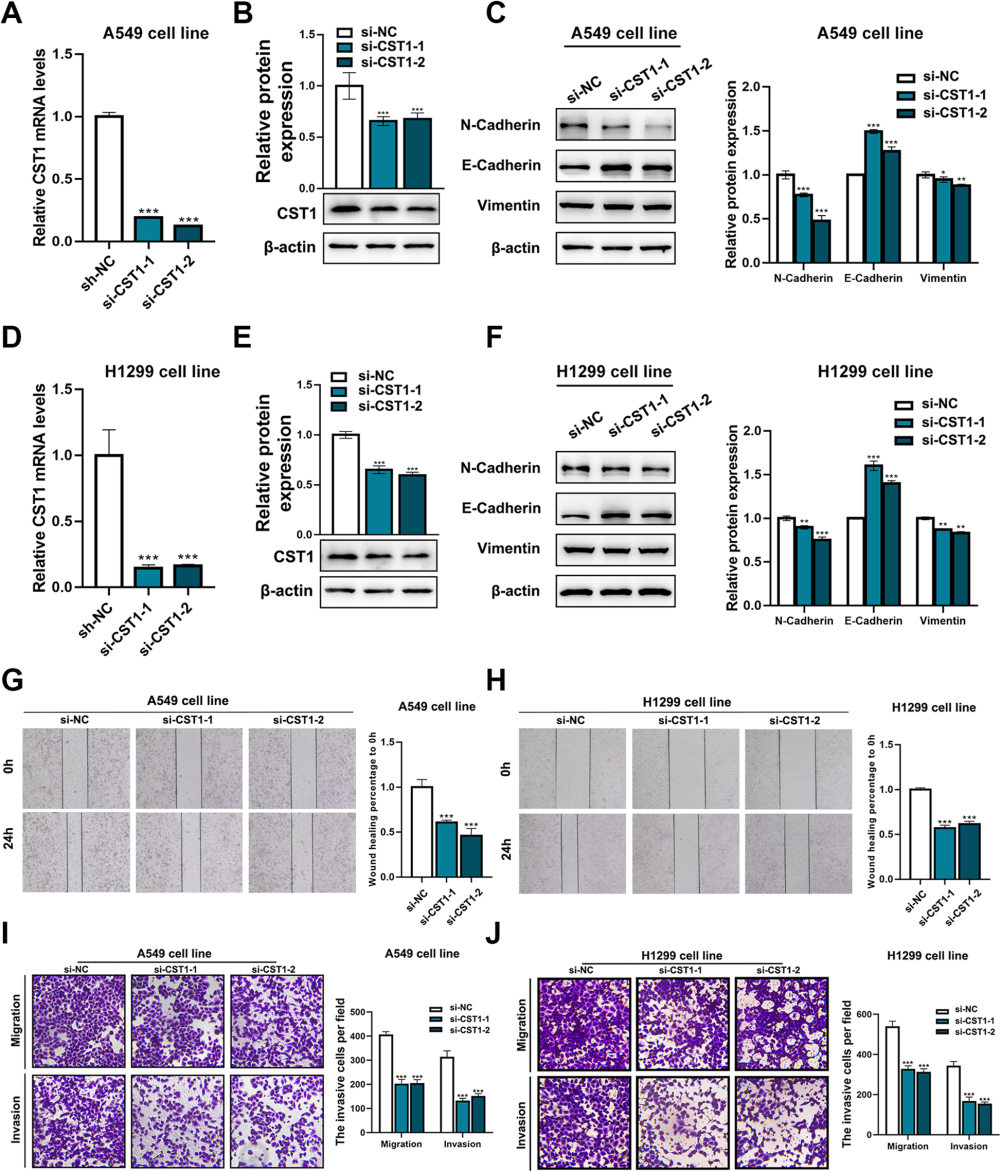
Knockdown of CST1 inhibits EMT in A549 and H1299 cells. **a**, **d** The mRNA levels of CST1 in CST1-silenced A549 and H1299 cells were detected by q-PCR. **b**, **e** The protein levels of CST1 in CST1-silenced A549 and H1299 cells were detected by western blotting. **c**, **f** The expression of EMT markers was detected by western blotting. **g**, **h** Wound-healing migration assays of CST1-silenced A549 and H1299 cells as well as control cells, which were treated as described above. **i**-**j** CST1-silenced A549 and H1299 cells were allowed to migrate through an 8-μM-pore size Transwell insert. Knockdown of CST1 inhibited the migration and invasion abilities of A549 and H1299 cells. Migrated and invaded cells were stained and counted in at least three microscopic fields. *p < 0.05, **p < 0.01 ***p < 0.001. (The blots were cutted prior to hybridisation with antibodies)

Moreover, we constructed A549 and H1299 cell lines with CST1 silencing. Real-time fluorescence quantification (Fig. 9a, d) and western blot (Fig. 9b, e) were utilized to detect the expression level of CST1 in the silenced cells. The results showed that CST1 was successfully knocked down. EMT-associated markers were detected in CST-silenced A549 and H1299 cell lines, revealing decreased levels of N-cadherin and Vimentin and an increased level of E-cadherin (Fig. 9c, f). We used the scratch assay to verify that the migration capacities of si-CST1–1 and si-CST1–2 cells were decreased compared with that of si-NC cells (Fig. 9g, h). Transwell experiments verified the decreased migration and invasion abilities of the CST1-silenced A549 and H1299 cell lines (Fig. 9i, j).

## Discussion

Lung adenocarcinoma is a highly heterogeneous and aggressive disease with extremely high morbidity and mortality. Therefore, finding valuable biomarkers for the prognostic assessment of lung adenocarcinoma plays an important role in lung adenocarcinoma therapy [35]. Recently, CST1 has received increased research attention as a member of the cystatin family of cysteine protease inhibitors [36]. However, the mechanisms of its aberrant expression in cancer and its biological behavior in cancer remain unclear. By comprehensively assessing the expression, clinicopathological significance, prognostic value, and functional impact of CST1 in LUAD, we found that CST1 is expressed at extremely high levels in LUAD patients and associated with poor prognosis. We revealed that CST1 is capable of promoting EMT, migration and invasion in LUAD, providing a solid basis for further downstream pathway studies.

CST1 has been shown to be highly expressed in a wide range of cancers, including gastric [37], breast [38], colon [9], thyroid [39], liver [10], and esophageal [40] cancers, and shown to be related to poor prognosis [39]. However, its role in LUAD has rarely been reported. In this study, we analyzed CST1 expression and prognostic information regarding LUAD using public databases such as TCGA-GTEx, TCGA, and GSE31210 and further validated that CST1 expression was notably higher in LUAD tissues than in paracancerous tissues. During cancer development [41], uncontrolled regulation of complex molecular networks often leads to abnormal gene expression [42]. Previous studies have shown that CST1 is strongly associated with the progression and aggressive metastasis of malignant tumors [43]. Although its role varies, CST1 contributes to hyperplasia, invasion, and metastasis in most malignancies. For example, Kim JT et al. identified CST1 and CST3 as having a 50% homologous binding domain [44]. CST1 can competitively bind CST3 with cathepsin B, and CST1-CST3 complexes have a higher affinity than CST3-cathepsin B complexes. This effect can counteract the inhibition of cathepsin B by CST3, thereby restoring the activity of cathepsin B in cells and promoting tumor progression. Yanfang Liu et al. found that CST1, as an oncogene in ER+ breast cancer, is involved in ER+ breast cancer progression by regulating the ERα/PI3K/AKT/ERα loop pathway [38]. Nevertheless, the function of CST1 in LUAD progression and malignant metastasis is unclear. A retrospective analysis of 174 cases of non-small-cell lung cancer was conducted separately by experts from Sun Yat-sen University [45]. In that study, they mapped the risk ratio of recurrence and metastasis in patients with LUAD and found a significant difference between the high and low CST1 expression groups. (*P* < 0.004) Moreover, differential expression of CST1 has prognostic significance. (*P* = 0.004).

In this study, we identified significant high expression of CST1, a member of the CST superfamily, in lung adenocarcinoma based on expression profiling from TCGA and GEO databases. Our results were validated by qRT-PCR and WB assays in lung adenocarcinoma samples and cell lines. Further, we found that the expression level of CST1 was correlated with clinicopathological features. By using prognostic analysis of Kaplan-Meier Plotterr and PrognoScan database, we found that high expression of CST1 was associated with poor prognosis in lung adenocarcinoma patients. These results suggested that CST1 may act as a pro-oncogene affecting tumor progression. Based on mutation analysis of CST1, we explored that DNA copy number alteration was the most common form of mutation. Patients with CST1 mutations had significantly worse disease-free survival, suggesting that increased frequency of CST1 mutations may contribute to tumor progression. In addition, through the PPI network constructed using the GeneMANIA and STRING databases, we speculated that the CST superfamily may play a synergistic role in co-acting as cancer promoters.

Notably, the role of the CST superfamily in the tumor immune microenvironment has previously been reported [46–48]. Therefore, this study also delved into the impact of CST1 on the tumor immune microenvironment. The components of the tumor microenvironment interact with tumor cells, affecting tumor growth, metastasis and drug resistance. By using the MCPCounter algorithm, we investigated the effect of CST1 on immune infiltration abundance in 10 cell types. Excitingly, we found a strong association between CST1 and cells in the tumor microenvironment. CST1 was positively associated with tumor-associated fibroblasts (CAFs). CAFs have previously been reported to play an important role in cancer [49, 50]. CAFs promote tumor progression, invasion and metastasis by secreting multiple cytokines and metabolites that inhibit immune cell function [51]. In addition, CAFs shape the extracellular matrix, forming a barrier to drug and immune cell infiltration. Therefore, CST1-induced infiltration of CAFs may inhibit immune cells capacity and further promote cancer. Remarkably, CST1 was negatively associated with neutrophils and endothelial cells. Neutrophils play a crucial role in inflammatory response and host defense against microbial infection. Recent studies have shown that neutrophils are thought to be part of an immune response that regulates tumor growth and metastasis and play a role in anti-tumor effects in humans [52]. Therefore, a decrease in neutrophils abundance due to CST1 may indirectly reduce neutrophils’ tumor inhibition. In addition, researchers at the Max Planck Institute in Germany found that tumor cells kill specific endothelial cells in the walls of blood vessels [53]. This allowed tumor cells to pass through the endothelial cell barrier in the lung, leaving the blood vessels to build metastatic cancer. Therefore, whether CST1 promotes tumor metastasis by killing endothelial cells through a similar mechanism deserves further investigation. Apart from stromal cells, we also found a negative correlation between CST1 expression and NK cells. NK cells have a variety of functions that limit the growth and spread of cancer cells [54]. Upon entering the tumor’s immune microenvironment, NK cells can kill cancer cells through a “loss of self” mechanism. Taken together, these analyses revealed that CST1 may alter the tumor microenvironment and play a role in suppressing anti-tumor immunity. Therefore, it is of practical significance to explore immunotherapy or targeted therapy targeting CST1.

To further investigate the biological function of CST1, we performed GO and KEGG enrichment analyses and found that CST1 is primarily involved in EMT-associated signaling pathways, such as the PI3K-Akt signaling, Wnt signaling, ECM-receptor interaction, and TGF-beta signaling pathways. To further verify the results of enrichment analysis, we performed GSEA analysis, which revealed a correlation between EMT and CST1. In view of the consistent results obtained by enrichment analysis, we hypothesized that CST1 may play an important role in lung adenocarcinoma metastasis and invasion. Therefore, we decided to use experiments to further validate the results of our bioinformatics analyses. Experiments validated that overexpression of CST1 promotes LUAD cell migration and invasion and while knockdown of CST1 inhibits LUAD cell migration and invasion. To sum up, although there are some limitations, the findings of this study provide exciting new clues that require further focused research to elucidate specific mechanisms.

## Conclusions

In conclusion, our comprehensive bioinformatics analyses revealed that CST1 was associated with the prognosis and clinical characteristics of LUAD patients, and it may be a new prognostic biomarker. Functional enrichment analysis and experimental validation revealed that CST1 promotes epithelial-mesenchymal transition. This study provided new insight into the prognostic role of CST1 in LUAD with potential target values.

## Supplementary Information

**Additional file 1.**

**Additional file 2.**

**Additional file 3.**

## Data Availability

The data supporting the conclusions was downloaded from TCGA database (https://portal.gdc.cancer.gov/) and GEO database (https://www.ncbi.nlm.nih.gov/geo/) or from the corresponding author on reasonable request.

## References

[1] Anastasi A, Brown MA, Kembhavi AA, Nicklin MJ, Sayers CA, Sunter DC, Barrett AJ (1983). Cystatin, a protein inhibitor of cysteine proteinases. Improved purification from egg white, characterization, and detection in chicken serum. Biochem J.

[2] Jin L, Zhang Y, Li H, Yao L, Fu D, Yao X, Xu LX, Hu X, Hu G (2012). Differential secretome analysis reveals CST6 as a suppressor of breast cancer bone metastasis. Cell Res.

[3] Kopitar-Jerala N (2015). Innate Immune Response in Brain, NF-Kappa B Signaling and Cystatins. Front Mol Neurosci.

[4] Breznik B, Mitrovic A, Lah TT, Kos J (2019). Cystatins in cancer progression: More than just cathepsin inhibitors. Biochimie.

[5] Shamsi A, Bano B (2017). Journey of cystatins from being mere thiol protease inhibitors to at heart of many pathological conditions. Int J Biol Macromol.

[6] Zhang WP, Wang Y, Tan D, Xing CG (2020). Cystatin 2 leads to a worse prognosis in patients with gastric cancer. J Biol Regul Homeost Agents.

[7] Zhang YQ, Zhang JJ, Song HJ, Li DW (2017). Overexpression of CST4 promotes gastric cancer aggressiveness by activating the ELFN2 signaling pathway. Am J Cancer Res.

[8] Dai DN, Li Y, Chen B, Du Y, Li SB, Lu SX, Zhao ZP, Zhou AJ, Xue N, Xia TL (2017). Elevated expression of CST1 promotes breast cancer progression and predicts a poor prognosis. J Mol Med (Berl).

[9] Jiang J, Liu HL, Tao L, Lin XY, Yang YD, Tan SW, Wu B (2018). Let7d inhibits colorectal cancer cell proliferation through the CST1/p65 pathway. Int J Oncol.

[10] Cui Y, Sun D, Song R, Zhang S, Liu X, Wang Y, Meng F, Lan Y, Han J, Pan S (2019). Upregulation of cystatin SN promotes hepatocellular carcinoma progression and predicts a poor prognosis. J Cell Physiol.

[11] Oh BM, Lee SJ, Cho HJ, Park YS, Kim JT, Yoon SR, Lee SC, Lim JS, Kim BY, Choe YK (2017). Cystatin SN inhibits auranofin-induced cell death by autophagic induction and ROS regulation via glutathione reductase activity in colorectal cancer. Cell Death Dis.

[12] Ding J, Wang X, Gao J, Song T. Silencing of cystatin SN abrogates cancer progression and stem cell properties in papillary thyroid carcinoma. FEBS Open Bio. 2021. 10.1002/2211-5463.13221 PMC8329778 34102026

[13] Chen S, Liu Y, Zhang K, Chen L (2021). CST1 Promoted Gastric Cancer Migration and Invasion Through Activating Wnt Pathway. Cancer Manag Res.

[14] Sung H, Ferlay J, Siegel RL, Laversanne M, Soerjomataram I, Jemal A, Bray F (2021). Global Cancer Statistics 2020: GLOBOCAN Estimates of Incidence and Mortality Worldwide for 36 Cancers in 185 Countries. CA Cancer J Clin.

[15] Biswas D, Birkbak NJ, Rosenthal R, Hiley CT, Lim EL, Papp K, Boeing S, Krzystanek M, Djureinovic D, La Fleur L (2019). A clonal expression biomarker associates with lung cancer mortality. Nat Med.

[16] Stuelten CH, Parent CA, Montell DJ (2018). Cell motility in cancer invasion and metastasis: insights from simple model organisms. Nat Rev Cancer.

[17] Liu K, Yu Q, Li H, Xie C, Wu Y, Ma D, Sheng P, Dai W, Jiang H (2020). BIRC7 promotes epithelial-mesenchymal transition and metastasis in papillary thyroid carcinoma through restraining autophagy. Am J Cancer Res.

[18] Chin L, Andersen JN, Futreal PA (2011). Cancer genomics: from discovery science to personalized medicine. Nat Med.

[19] Warde-Farley D, Donaldson SL, Comes O, Zuberi K, Badrawi R, Chao P, Franz M, Grouios C, Kazi F, Lopes CT (2010). The GeneMANIA prediction server: biological network integration for gene prioritization and predicting gene function. Nucleic Acids Res.

[20] Szklarczyk D, Gable AL, Lyon D, Junge A, Wyder S, Huerta-Cepas J, Simonovic M, Doncheva NT, Morris JH, Bork P (2019). STRING v11: protein-protein association networks with increased coverage, supporting functional discovery in genome-wide experimental datasets. Nucleic Acids Res.

[21] Tang Z, Kang B, Li C, Chen T, Zhang Z (2019). GEPIA2: an enhanced web server for large-scale expression profiling and interactive analysis. Nucleic Acids Res.

[22] Brlek P, Kafka A, Bukovac A, Pecina-Slaus N (2021). Integrative cBioPortal Analysis Revealed Molecular Mechanisms That Regulate EGFR-PI3K-AKT-mTOR Pathway in Diffuse Gliomas of the Brain. Cancers (Basel).

[23] Gan J, Li Y, Meng Q (2020). Systematic Analysis of Expression Profiles and Prognostic Significance for FAM83 Family in Non-small-Cell Lung Cancer. Front Mol Biosci.

[24] Mizuno H, Kitada K, Nakai K, Sarai A (2009). PrognoScan: a new database for meta-analysis of the prognostic value of genes. BMC Med Genet.

[25] Kanehisa M, Furumichi M, Sato Y, Ishiguro-Watanabe M, Tanabe M (2021). KEGG: integrating viruses and cellular organisms. Nucleic Acids Res.

[26] The Gene Ontology C (2019). The Gene Ontology Resource: 20 years and still GOing strong. Nucleic Acids Res.

[27] Wu T, Hu E, Xu S, Chen M, Guo P, Dai Z, Feng T, Zhou L, Tang W, Zhan L (2021). clusterProfiler 4.0: A universal enrichment tool for interpreting omics data. Innovation (N Y).

[28] Cosset E, Ilmjarv S, Dutoit V, Elliott K, von Schalscha T, Camargo MF, Reiss A, Moroishi T, Seguin L, Gomez G (2017). Glut3 addiction is a druggable vulnerability for a molecularly defined subpopulation of glioblastoma. Cancer Cell.

[29] Lentini JM, Alsaif HS, Faqeih E, Alkuraya FS, Fu D (2020). DALRD3 encodes a protein mutated in epileptic encephalopathy that targets arginine tRNAs for 3-methylcytosine modification. Nat Commun.

[30] Schmittgen TD, Livak KJ (2008). Analyzing real-time PCR data by the comparative C(T) method. Nat Protoc.

[31] Melgar-Lesmes P, Luquero A, Parra-Robert M, Mora A, Ribera J, Edelman ER, Jimenez W (2018). Graphene-dendrimer nanostars for targeted macrophage overexpression of metalloproteinase 9 and hepatic fibrosis precision therapy. Nano Lett.

[32] Duchamp de Lageneste O, Julien A, Abou-Khalil R, Frangi G, Carvalho C, Cagnard N, Cordier C, Conway SJ, Colnot C (2018). Periosteum contains skeletal stem cells with high bone regenerative potential controlled by Periostin. Nat Commun.

[33] Kosinsky RL, Zerche M, Saul D, Wang X, Wohn L, Wegwitz F, Begus-Nahrmann Y, Johnsen SA (2020). USP22 exerts tumor-suppressive functions in colorectal cancer by decreasing mTOR activity. Cell Death Differ.

[34] Tan WCC, Nerurkar SN, Cai HY, Ng HHM, Wu D, Wee YTF, Lim JCT, Yeong J, Lim TKH (2020). Overview of multiplex immunohistochemistry/immunofluorescence techniques in the era of cancer immunotherapy. Cancer Commun (Lond).

[35] Zhang XW, Li L, Hu WQ, Hu MN, Tao Y, Hu H, Miao XK, Yang WL, Zhu Q, Mou LY (2022). Neurokinin-1 receptor promotes non-small cell lung cancer progression through transactivation of EGFR. Cell Death Dis.

[36] Wu D, Yan B, Wang Y, Wang C, Zhang L (2021). Prognostic and pharmacologic value of cystatin SN for chronic rhinosinusitis with nasal polyps. J Allergy Clin Immunol.

[37] Choi EH, Kim JT, Kim JH, Kim SY, Song EY, Kim JW, Kim SY, Yeom YI, Kim IH, Lee HG (2009). Upregulation of the cysteine protease inhibitor, cystatin SN, contributes to cell proliferation and cathepsin inhibition in gastric cancer. Clin Chim Acta.

[38] Liu Y, Ma H, Wang Y, Du X, Yao J (2019). Cystatin SN affects cell proliferation by regulating the ERalpha/PI3K/AKT/ERalpha loopback pathway in breast cancer. Onco Targets Ther.

[39] Liu Y, Liao L, An C, Wang X, Li Z, Xu Z, Liu J, Liu S (2021). alpha-Enolase Lies Downstream of mTOR/HIF1alpha and Promotes Thyroid Carcinoma Progression by Regulating CST1. Front Cell Dev Biol.

[40] Wang J, Yu L, Sun Y, Zhang L, Tu M, Cai L, Yin X, Pan X, Wang T, Huang Y (2021). Development and Evaluation of Serum CST1 Detection for Early Diagnosis of Esophageal Squamous Cell Carcinoma. Cancer Manag Res.

[41] Masuda M, Uno Y, Ohbayashi N, Ohata H, Mimata A, Kukimoto-Niino M, Moriyama H, Kashimoto S, Inoue T, Goto N (2016). TNIK inhibition abrogates colorectal cancer stemness. Nat Commun.

[42] Kuenzi BM, Ideker T (2020). A census of pathway maps in cancer systems biology. Nat Rev Cancer.

[43] Jiang J, Liu HL, Liu ZH, Tan SW, Wu B (2015). Identification of cystatin SN as a novel biomarker for pancreatic cancer. Tumour Biol.

[44] Kim JT, Lee SJ, Kang MA, Park JE, Kim BY, Yoon DY, Yang Y, Lee CH, Yeom YI, Choe YK (2013). Cystatin SN neutralizes the inhibitory effect of cystatin C on cathepsin B activity. Cell Death Dis.

[45] Cao X, Li Y, Luo RZ, Zhang L, Zhang SL, Zeng J, Han YJ, Wen ZS (2015). Expression of Cystatin SN significantly correlates with recurrence, metastasis, and survival duration in surgically resected non-small cell lung cancer patients. Sci Rep.

[46] Jakos T, Pislar A, Jewett A, Kos J (2019). Cysteine Cathepsins in Tumor-Associated Immune Cells. Front Immunol.

[47] Kos J, Nanut MP, Prunk M, Sabotic J, Dautovic E, Jewett A (2018). Cystatin F as a regulator of immune cell cytotoxicity. Cancer Immunol Immunother.

[48] Laurent-Matha V, Huesgen PF, Masson O, Derocq D, Prebois C, Gary-Bobo M, Lecaille F, Rebiere B, Meurice G, Orear C (2012). Proteolysis of cystatin C by cathepsin D in the breast cancer microenvironment. FASEB J.

[49] Biffi G, Tuveson DA (2021). Diversity and Biology of Cancer-Associated Fibroblasts. Physiol Rev.

[50] Kalluri R (2016). The biology and function of fibroblasts in cancer. Nat Rev Cancer.

[51] Goulet CR, Champagne A, Bernard G, Vandal D, Chabaud S, Pouliot F, Bolduc S (2019). Cancer-associated fibroblasts induce epithelial-mesenchymal transition of bladder cancer cells through paracrine IL-6 signalling. BMC Cancer.

[52] Teijeira A, Garasa S, Ochoa MC, Villalba M, Olivera I, Cirella A, Eguren-Santamaria I, Berraondo P, Schalper KA, de Andrea CE (2021). IL8, Neutrophils, and NETs in a Collusion against Cancer Immunity and Immunotherapy. Clin Cancer Res.

[53] Strilic B, Yang L, Albarran-Juarez J, Wachsmuth L, Han K, Muller UC, Pasparakis M, Offermanns S (2016). Tumour-cell-induced endothelial cell necroptosis via death receptor 6 promotes metastasis. Nature.

[54] Xia J, Minamino S, Kuwabara K (2020). CAR-expressing NK cells for cancer therapy: a new hope. Biosci Trends.

