# Supplementary material for: Cystatin SN promotes epithelial-mesenchymal transition and serves as a prognostic biomarker in lung adenocarcinoma
Source: BMC Cancer. 2022 May 30;22:589. doi: 10.1186/s12885-022-09685-z (PMC9150371; doi:10.1186/s12885-022-09685-z)
Supplement: Supplementary file 1 — Additional file 1. [file 12885_2022_9685_MOESM1_ESM.docx]

| **GO** | **Category** | **Description** | ***p* value** | **Count** |
| --- | --- | --- | --- | --- |
|  |  |  |  |  |
| GO:0043062 | GO Biological Processes | extracellular structure organization | 6.71899E-31 | 62 |
| GO:0030198 | GO Biological Processes | extracellular matrix organization | 1.92723E-33 | 61 |
| GO:0001503 | GO Biological Processes | ossification | 2.30394E-14 | 40 |
| GO:0050673 | GO Biological Processes | epithelial cell proliferation | 6.79758E-09 | 33 |
| GO:0050900 | GO Biological Processes | leukocyte migration | 1.89013E-07 | 33 |
| GO:0030111 | GO Biological Processes | regulation of Wnt signaling pathway | 3.03155E-10 | 32 |
| GO:0048568 | GO Biological Processes | embryonic organ development | 1.73048E-08 | 32 |
| GO:0061448 | GO Biological Processes | connective tissue development | 8.63511E-13 | 31 |
| GO:0090287 | GO Biological Processes | regulation of cellular response to growth factor stimulus | 5.13681E-12 | 31 |
| GO:0060070 | GO Biological Processes | canonical Wnt signaling pathway | 7.59872E-10 | 30 |
| GO:0062023 | GO Cellular Components | collagen-containing extracellular matrix | 7.60513E-39 | 70 |
| GO:0005788 | GO Cellular Components | endoplasmic reticulum lumen | 8.07121E-10 | 29 |
| GO:0030055 | GO Cellular Components | cell-substrate junction | 4.57813E-07 | 29 |
| GO:0005925 | GO Cellular Components | focal adhesion | 1.0333E-06 | 28 |
| GO:0005924 | GO Cellular Components | cell-substrate adherens junction | 1.19502E-06 | 28 |
| GO:0031252 | GO Cellular Components | cell leading edge | 0.000182345 | 23 |
| GO:0034774 | GO Cellular Components | secretory granule lumen | 0.001089903 | 18 |
| GO:0060205 | GO Cellular Components | cytoplasmic vesicle lumen | 0.001932177 | 18 |
| GO:0031983 | GO Cellular Components | vesicle lumen | 0.001995317 | 18 |
| GO:0005581 | GO Cellular Components | collagen trimer | 3.445E-10 | 16 |
| GO:0005201 | GO Molecular Functions | extracellular matrix structural constituent | 1.53004E-27 | 39 |
| GO:0048018 | GO Molecular Functions | receptor ligand activity | 1.29856E-08 | 35 |
| GO:0050839 | GO Molecular Functions | cell adhesion molecule binding | 3.04241E-07 | 33 |
| GO:0005539 | GO Molecular Functions | glycosaminoglycan binding | 9.86844E-11 | 26 |
| GO:0004175 | GO Molecular Functions | endopeptidase activity | 0.000993895 | 22 |
| GO:0005125 | GO Molecular Functions | cytokine activity | 5.86017E-07 | 20 |
| GO:0005178 | GO Molecular Functions | integrin binding | 6.18559E-10 | 19 |
| GO:0019838 | GO Molecular Functions | growth factor binding | 7.78327E-09 | 18 |
| GO:0005126 | GO Molecular Functions | cytokine receptor binding | 0.000284039 | 18 |
| GO:0033218 | GO Molecular Functions | amide binding | 0.003393721 | 18 |

**Additional file 1: Table S1** TOP 30 GO functional enrichment analysis of CST1 co-expressed genes in LUAD.
