# Supplementary material for: Cystatin SN promotes epithelial-mesenchymal transition and serves as a prognostic biomarker in lung adenocarcinoma
Source: BMC Cancer. 2022 May 30;22:589. doi: 10.1186/s12885-022-09685-z (PMC9150371; doi:10.1186/s12885-022-09685-z)
Supplement: Supplementary file 2 — Additional file 2. [file 12885_2022_9685_MOESM2_ESM.docx]

**Additional file 2: Table S2** Top 20 KEGG functional enrichment analysis of CST1 co-expressed genes in LUAD.

| **Term** | **Category** | **Description** | **Count** | ***p* value** | **p.adjust** |
| --- | --- | --- | --- | --- | --- |
| hsa05205 | KEGG Pathway | Proteoglycans in cancer | 21 | 3.17332E-08 | 7.13998E-06 |
| hsa05165 | KEGG Pathway | Human papillomavirus infection | 20 | 0.000206558 | 0.003450438 |
| hsa04151 | KEGG Pathway | PI3K-Akt signaling pathway | 20 | 0.000496773 | 0.00745159 |
| hsa04510 | KEGG Pathway | Focal adhesion | 18 | 2.34484E-06 | 0.000105518 |
| hsa04060 | KEGG Pathway | Cytokine-cytokine receptor interaction | 16 | 0.002756562 | 0.026966372 |
| hsa05225 | KEGG Pathway | Hepatocellular carcinoma | 15 | 1.74447E-05 | 0.000490632 |
| hsa04390 | KEGG Pathway | Hippo signaling pathway | 14 | 3.39749E-05 | 0.000849372 |
| hsa04310 | KEGG Pathway | Wnt signaling pathway | 14 | 4.19528E-05 | 0.000943937 |
| hsa04974 | KEGG Pathway | Protein digestion and absorption | 13 | 1.42686E-06 | 8.94638E-05 |
| hsa05226 | KEGG Pathway | Gastric cancer | 13 | 8.16041E-05 | 0.001669175 |
| hsa04512 | KEGG Pathway | ECM-receptor interaction | 12 | 1.59047E-06 | 8.94638E-05 |
| hsa05414 | KEGG Pathway | Dilated cardiomyopathy | 12 | 4.06307E-06 | 0.000152365 |
| hsa05217 | KEGG Pathway | Basal cell carcinoma | 11 | 3.42779E-07 | 3.85626E-05 |
| hsa05410 | KEGG Pathway | Hypertrophic cardiomyopathy | 11 | 1.28738E-05 | 0.0004138 |
| hsa05224 | KEGG Pathway | Breast cancer | 11 | 0.001064648 | 0.014090936 |
| hsa04530 | KEGG Pathway | Tight junction | 11 | 0.003233622 | 0.030315202 |
| hsa04350 | KEGG Pathway | TGF-beta signaling pathway | 10 | 0.000106887 | 0.002004138 |
| hsa05418 | KEGG Pathway | Fluid shear stress and atherosclerosis | 10 | 0.002392725 | 0.025636342 |
| hsa04670 | KEGG Pathway | Leukocyte transendothelial migration | 9 | 0.00208259 | 0.024337365 |
| hsa05412 | KEGG Pathway | Arrhythmogenic right ventricular cardiomyopathy | 8 | 0.00061856 | 0.008698496 |
