# Supplementary material for: Cystatin SN promotes epithelial-mesenchymal transition and serves as a prognostic biomarker in lung adenocarcinoma
Source: BMC Cancer. 2022 May 30;22:589. doi: 10.1186/s12885-022-09685-z (PMC9150371; doi:10.1186/s12885-022-09685-z)
Supplement: Supplementary file 3 — Additional file 3. [file 12885_2022_9685_MOESM3_ESM.docx]

**Additional file 3: Table S3** GSEA analysis of CTS1 related enriched gene set with the high and low expression of CST1.

NES: normalized enrichment score; FDR: false discovery rate. Gene sets with p.adjust<0.05 and FDR<0.25 are considered as significant.

| **MsigDB collection** | **Description** | **NES** | **p.adjust** | **FDR** |
| --- | --- | --- | --- | --- |
| h.all.v7.2.symbols.gmt [Hallmarks] | HALLMARK_EPITHELIAL_MESENCHYMAL_TRANSITION | 2.04 | 0.004 | 0.011 |
|  | HALLMARK_P53_PATHWAY | 2.05 | 0.000 | 0.019 |
|  | HALLMARK_COAGULATION | 1.92 | 0.000 | 0.032 |
|  | HALLMARK_NOTCH_SIGNALING | 1.83 | 0.008 | 0.069 |
|  | HALLMARK_APICAL_JUNCTION | 1.80 | 0.012 | 0.066 |
|  | HALLMARK_MYOGENESIS | 1.79 | 0.012 | 0.062 |
|  | HALLMARK_APOPTOSIS | 1.75 | 0.008 | 0.069 |
|  | HALLMARK_ANGIOGENESIS | 1.70 | 0.020 | 0.090 |
|  | HALLMARK_IL2_STAT5_SIGNALING | 1.51 | 0.049 | 0.241 |
